# School Nurses’ Experiences of the School-Based HPV Vaccination Program – A Decade Later

**DOI:** 10.1177/10598405251374726

**Published:** 2025-09-08

**Authors:** Ida Enskär, Tryggve Nevéus, Karin Enskär, Carolina Källqvist, Maria Grandahl

**Affiliations:** 1 Department of Women's and Children's Health, 8097 Uppsala University, Akademiska Hospital, Uppsala, Sweden

**Keywords:** attitudes, experiences, HPV vaccination, knowledge, school-based immunization program, school nurse

## Abstract

This study investigated Swedish school nurses’ experiences, self-perceived knowledge, and attitudes towards HPV vaccination. A cross-sectional repeated questionnaire study was conducted. The results were compared to a previous study conducted in 2016. Additionally, open-ended responses were thematically analysed. A total of 344 nurses participated. Overall, more participants reported good self-perceived knowledge and favourable attitudes towards HPV vaccination compared to 2016. Still, nearly half expressed a need for further education. The thematic analysis revealed barriers including lack of training, inconsistent guidelines for addressing vaccine hesitancy and ensuring children's participation in decision-making, limited access to diverse information materials, and time constraints related to vaccination tasks. Since school nurses in Sweden are responsible for all aspects of school-aged vaccinations within the national immunization program, it is essential that they receive support to feel motivated and confident in addressing vaccine hesitancy. Continued education and the implementation of uniform guidelines are therefore necessary.

Human papillomavirus (HPV) vaccine prevents HPV-related cancers in both men and women, especially cervical cancer which is the second most common cancer among women of reproductive age (Sung et al., 2021). Unfortunately, vaccination rates remain suboptimal in most countries, meaning they fall below the levels needed to achieve herd immunity and meet public health targets (Shapiro, 2022). Consequently, there is a risk of not achieving the World Health Organization's (WHO) goal to eliminate cervical cancer - a strategy in which one of the three central targets is to reach 90% HPV vaccination coverage among girls by 2030 (World Health Organization, 2020a).

HPV vaccination has been offered free-of-charge in Sweden through the national school-based childhood immunization program (NIP) since 2012 for girls. In 2020, the gender-neutral vaccination program was introduced, meaning that all children in fifth grade (ages 10–12) are now offered HPV vaccination. In Sweden, compliance with early childhood vaccinations is high but acceptance of the HPV vaccination is lower: in 2023, 88% of all girls and 83% of all boys aged 12 were fully vaccinated with two doses (The Public Health Agency of Sweden, 2022; The Public Health Agency of Sweden, 2023).

To decline or postpone recommended vaccines despite available vaccination services, referred to as vaccine hesitancy, is considered a top-ten threat to global health by the WHO (World Health Organization, 2019a). This is a complex issue with varying impact in different sub-populations. Reasons for hesitancy may vary over time and type of vaccines (MacDonald, 2015). Specific challenges for the HPV vaccination concern its protection against a sexually transmitted infection (STI) and the young age (9–14) for recommended vaccination (Grandahl & Nevéus, 2021; López et al., 2020). The erroneous idea that vaccination may encourage sexual activity is commonly reported (López et al., 2020). Among boys and their parents, there is a misconception that HPV is an exclusively female problem (Grandahl & Nevéus, 2021).

One of the most important factors for the parental decision to vaccinate their children is trust in healthcare providers’ (HCPs) recommendations (Lin et al., 2021). In Sweden, nurses play a crucial role in parental acceptance of childhood vaccinations by building trust in the national vaccination program from an early stage, and support informed parental decision-making (Appelqvist et al., 2023). School nurses i.e., registered nurses with a specialist degree, are responsible for all aspects of administering recommended vaccinations to school-aged children and are considered the most reliable source of HPV vaccine information by Swedish school children (Enskär et al., 2024; Grandahl et al., 2019). The school nurse responsibilities include informing both children and parents, administering the vaccines, and offering vaccinations to unvaccinated children. Consequently, school nurses, the HCPs in this setting, play a key role in the success of the school-based vaccination program. However, HCPs may lack adequate knowledge, training, and confidence to deliver strong recommendations for HPV vaccination. Moreover, their own attitudes and vaccine hesitancy—shaped by concerns about safety, efficacy, or anticipated parental resistance—can significantly influence whether and how they recommend the vaccine (Lin et al., 2021). In 2013, following the introduction of the school-based HPV vaccination program, the research group performed a cross-sectional study to investigate school nurses’ knowledge of, attitudes towards and experiences of HPV vaccinations (Grandahl et al., 2014). In 2016 a repeated study was conducted (Grandahl et al., 2017). Generally, school nurses reported more favorable attitudes towards the HPV vaccine in 2016 compared to 2013, but still 56% stated they needed more knowledge. In 2016 a larger proportion of school nurses (90%) had been contacted by parents who had concerns about the vaccine, compared to 76% in 2013 (Grandahl et al., 2017). The increase of parental concerns in 2016 was likely due to extensive media debates and spread of misinformation about the safety of the vaccine at the time. These events had massive implications for well-functioning vaccination programs in other countries (Hanley et al., 2015; Suppli et al., 2018). Recently, we have experienced how the COVID-19 pandemic has caused setbacks in vaccination rates worldwide (Casey et al., 2024; Karafillakis et al., 2022). These instances highlight the importance of continuously monitoring vaccination programs. Given the crucial role of school nurses in HPV vaccinations, it is important to follow up on their experiences to ensure resilience of the school-based vaccination program. Therefore, this study aimed to investigate their experiences of the school-based HPV vaccination program. More specifically, to investigate:

1. If more school nurses had favorable attitudes toward HPV vaccination in 2024 compared to the previous study in 2016
2. If more school nurses considered that they had good knowledge about HPV vaccination in 2024 compared to the previous study in 2016
3. School nurses’ experiences of providing information about HPV vaccination
4. School nurses’ experiences of barriers and facilitators for HPV vaccination

## Methods

### Study Design

This was a repeated cross-sectional questionnaire study.

### Ethical Considerations

The study was conducted in accordance with the Declaration of Helsinki and was approved by the Swedish Ethical Review Authority (dnr: 2023–01583–01). Informed consent was obtained from all participants.

### Setting

In Sweden, HPV vaccination is mainly offered in fifth grade (children aged 10 to 12) in a two-dose scheme and requires parental consent. Unvaccinated children can get vaccinated free-of-charge through the school health services until the age of 18. In 2020, the Convention on the Rights of the Child was elevated to Swedish law [2018 : 1197]. The law does not state any age at when children are allowed to self-consent to healthcare decisions, but HCPs should evaluate the child's maturity on a case-by-case basis.

### Recruitment of Participants

School nurses working with HPV vaccinations, currently employed in public or private schools, were eligible to participate. There is no official employment data on how many school nurses are working with HPV vaccination in Sweden. However, in total there were total 3,060 school nurses employed (part- or full time) in state driven primary and secondary schools in 2023 (The Swedish Association of Local Authorities and Regions, 2025). Participants were recruited in collaboration with the National Board of Swedish School Nurses (Riksföreningen för skolsköterskor, n.d). Study information was provided at their annual national congress in 2023, with approximately 1500 attending school nurses, as well as through their social media platforms. Additionally, we provided information about the study to the head of school health services and/or directly to eligible school nurses in each of the 290 municipalities. Participants were able to participate through Research Electronic Data Capture (REDCap) tools hosted at the university, or having the survey sent out in paper format (Harris et al., 2009; Harris et al., 2019). Written consent was attained from all participants. Two reminder emails were sent out if the school nurse had consented but not completed the questionnaire. Data collection started in spring 2023 and ended in spring 2024.

### Questionnaire

The study-specific questionnaire used in the 2013 and 2016 study (Grandahl et al., 2014; Grandahl et al., 2017) was based on findings from previous studies clinical experience and input from experts in relevant fields. The original questionnaire's validity had been tested by a group of ten school nurses. In 2024, some of the gendered questions from the 2016 questionnaire were removed or re-phrased due to irrelevance or relating to recent changes of the NIP. Based on previous qualitative studies (Enskär et al., 2023; Enskär et al., 2024) questions about school nurses’ experiences were added. Furthermore, questions regarding children's participation in the vaccination decision were added to reflect recent legislation changes. The new questionnaire was again tested on three head school nurses before use.

In total, the final questionnaire consisted of 35 questions. Questions about participants’ background characteristics (n = 13) included age, sex, years of work experience, nursing specialist degree (pediatric nursing, public health or district nursing, school health nursing, other or none), school area (urban, rural or metropolitan), estimated number of children offered vaccination, and vaccination coverage at school level.

The questionnaire compromised the following main concepts:

- Attitudes toward HPV vaccination (n = 2)
- Self-perceived knowledge about HPV vaccination (n = 5)
- Experiences of providing information about HPV vaccination (n = 12)

The concepts of attitudes and self-perceived knowledge consisted of statements followed by a multiple-choice four-point scale “Strongly agree” “Partially agree”, “Partially disagree” or “Strongly disagree”. Questions regarding experiences were followed by different multiple-choice answers, further described in Table 4.

Finally, there were five free text answers to questions concerning: other common reasons for parents to decline or postpone HPV vaccination, the most frequently received question from children about HPV vaccination, strategies used when communicating with hesitant parents, experienced difficulties with HPV vaccinations, and additional comments about HPV vaccinations. The 2024 questionnaire is provided as a supplementary file (S1).

### Data Analysis

#### Quantitative Data Analysis

First, the quantitative data were described and comparisons were made between the 2024 and 2016 data. Differences were calculated using Mann-Whitney U test for ordinal variables, and Pearson's χ² or Fisher's Exact test for nominal variables. Independent sample t-test was used to compare scale variables between the two studies. Data were analyzed using SPSS software (28.0) and p-values of < 0.05 were considered statistically significant. The following variables were dichotomized: biological sex (man/woman), specialist degree (yes/no), and geographical area (rural/urban). Missing data was excluded from analysis.

Favorable attitudes toward HPV vaccination were defined as agreement with the following statements: the introduction of the vaccine to the NIP was appropriate, to have confidence in authorities’ decisions regarding the vaccination, that vaccination may increase awareness of sexually transmitted infections, and that children who initially decline should vaccination should be offered again. Additionally, disagreement with statements of common misconceptions about HPV vaccination – vaccination can cause serious side effects, vaccination may reduce participation in cervical screening, vaccination may result in increased number of sexual partners, and vaccination may result in decreased condom use – was considered indicative of a favorable attitude.

The 2024 data regarding school nurses’ experiences of providing information were presented in frequencies (n) and percentages (%). Data from the open-ended question concerning the most frequently received questions from children were sorted according to the topics and then quantified.

#### Qualitative Data Analysis of Open-Ended Questions

The COM-B model was used for a deductive thematic analysis of the qualitative data derived from open-ended questions in the 2024 questionnaire (Braun & Clarke, 2006). The WHO has adopted the COM-B model to assist authorities in identifying and analyzing factors that affect vaccination behaviors (World health Organization, 2019b). The model is based on the Behavior Change Wheel which allows for the identification of evidence-based intervention functions (Michie et al., 2011). It can be described as an interactional framework in which the behavioral determinants Capability (C), Opportunity (O) and Motivation (M), are needed to be in place for a behavior (B) to occur. Capabilities refer to having the required knowledge, skills and abilities; opportunities include contextual determinants impacting or making a behavior possible, such as sufficient time and resources; whereas motivation refers to internal processes influencing whether a person wants to perform a behavior. Motivational factors can be reflective (planning and evaluation of past experiences), or automatic (impulses, desires and inhibitions) (Michie et al., 2011). The use of the model allows to identify both individual and contextual determinants that can facilitate or hinder behaviors related to HPV vaccination. The steps of the thematic analysis are summarized below.

1. Searching for theory-driven themes: After familiarization with the data, text units were coded according to the pre-defined themes of the COM-B model's behavioral determinants. Units were then grouped together under the themes identified.
2. Identifying barriers and facilitators: sub-themes describing barriers and facilitating factors within each theme were identified.
3. Distinguishing between behaviors related to the varied tasks involved in school nurses’ work with vaccinations (B): The sub-themes were sorted according to the different behaviors described in the text units.

The analysis was an iterative process going back and forth between the original texts and the themes and sub-themes.

## Results

### Participants’ Background Characteristics

In total, 344 school nurses, representing all geographical regions (n = 21), and employed in 163/290 different municipalities participated in the present study. The sample size was lower in 2024 (n = 344) compared to 2016 (n = 736) but background characteristics were similar, as shown in Table 1. More school nurses reported a school vaccination coverage above 91% compared to 2016 (66% vs 43%, p < 0.001). Participants mainly vaccinated children in fifth grade (80%), while 10% vaccinated in lower-secondary school and 10% in upper-secondary school.

**Table 1.** Background Characteristics of Participating School Nurses 2024 (n = 344) and 2016 (n = 736).

	2024 (n = 344)		2016 (n = 736)		
	Mean [SD] or n (%)		Mean [SD] or n (%)		*p-value*
Age, years	49.4	[9.2]	51.4	[8.5]	0.001
Years as a school nurse	8.4	[7.0]	10.0	[7.8]	0.001
Work experience of HPV vaccination (years)	6.9	[4.5]	X	X	
					
Sex					0.246^ab^
Women	325	(94.5)	715	(97.1)	
Men	6	(1.8)	7	(1.0)	
Other/not stated	13	(3.7)	8	(1.1)	
Specialist nursing Education*					0.5^c^
Public health or district nursing	119	(35.6)	336	(45.7)	
Paediatric nursing	99	(29.6)	225	(30.6)	
School health	28	(8.4)	53	(7.2)	
Other (intensive care, anaesthesia, midwifery, etc.)	80	(24.0)	25	(3.4)	
None	8	(2.4)	21	(2.9)	
Workplace					0.5
Public school	304	(91.6)	650	(88.3)	
Private school	28	(8.4)	70	(9.5)	
Geographical area of the school/schools*					0.014^d^
Urban	187	(56.3)	337	(46.7)	
Rural	66	(19.9)	194	(26.9)	
Greater metropolitan area	79	(23.8)	191	(26.5)	
Number of children vaccinated this year (approximately)					
≤ 10	34	(10.5)	X	X	
11–60	167	(50.0)	X	X	
≥ 60	132	(39.5)	X	X	
Vaccination coverage at the school or schools of employment have been approximately					
≤ 70%	3	(0.9)	74	(10.1)	< 0.001†
71–80%	9	(2.6)	103	(14.1)	
81–90%	221	(21.5)	193	(26.4)	
91–100%	72	(66.0)	315	(43.2)	
Don’t know	30	(9.0)	45	(6.2)	

* Multiple answers could be applicable.

X Question not included in questionnaire.

^a^ Fisher exact test due to less than 5 expected counts in one cell.

^b^ Variable grouped by biological sex: man or woman.

^c^ Grouped: having a specialist degree or lack specialist degree.

^d^ Grouped: rural or urban or greater metropolitan area.

^†^Mann-Whitney U test.

### School Nurses’ Attitudes Toward HPV Vaccination: A Comparison Between 2016 and 2024

In general, more participants reported favorable attitudes to the national HPV vaccination in 2024 in comparison with 2016. Detailed results are presented in Table 2. There were statistically significant differences in all attitude statements except for “*HPV vaccine is more painful than other vaccines”,* and *“Fear of needles is a problem”* (see Table 2). For example, more school nurses strongly agreed to the statements: *it's appropriate that HPV vaccination was introduced in the national childhood vaccination program* (99% in 2024 vs 86% in 2016, p = 0.001), and *having confidence in the authority's decisions about HPV vaccination* (96% in 2024 vs 67% in 2016, p < 0.001). Additionally, more school nurses disagreed to statements of known misconceptions. Still, a quarter partially agreed to the statement that HPV vaccination may reduce participation in cervical screening and 6% that vaccination may result in an increased number of sexual partners.

**Table 2.** Attitudes Towards HPV Vaccination Among Participating School Nurses in 2024 (n = 344) and 2016 (n = 736).

	Strongly agree		Partially Agree		Partially disagree		Strongly disagree		*p-value*†
	n (%)		n (%)		n (%)		n (%)		
	2024	2016	2024	2016	2024	2016	2024	2016	
It is appropriate that HPV vaccination was introduced in the national childhood vaccination program	336 (99.1)	629 (85.5)	3 (0.9)	88 (12.0)	0	13 (1.8)	0	2 (0.3)	0.001
The HPV vaccine can cause serious side effects	3 (0.9)	30 (4.1)	14 (4.2)	149 (20.6)	101 (30.5)	298 (41.2)	213 (64.4)	247 (34.1)	< 0.001
The HPV vaccine is more painful than other vaccines	64 (19.2)	115 (15.6)	102 (30.5)	247 (33.6)	53 (15.9)	120 (16.3)	115 (34.4)	244 (33.2)	0.75
Fear of needles is a problem	54 (16.2)	132 (17.9)	172 (51.5)	328 (44.6)	73 (21.8)	158 (21.5)	35 (10.5)	108 (14.7)	0.37
Those who decline vaccination should be offered the vaccination later	287 (85.4)	278 (37.8)	30 (8.9)	234 (31.8)	16 (4.8)	120 (16.3)	3 (0.9)	98 (13.3)	< 0.001
I have confidence in the authority's decisions about HPV vaccination	322 (96.4)	491 (66.7)	8 (2.4)	197 (26.8)	1 (0.3)	27 (3.7)	3 (0.9)	12 (1.2)	< 0.001
HPV vaccination may result in decreased condom use	0	34 (4.6)	48 (14.5)	271 (36.8)	123 (37.2)	216 (29.3)	160 (48.3)	203 (27.6)	< 0.001
HPV vaccination may result in increased number of sexual partners	0	12 (1.6)	19 (5.8)	138 (18.8)	83 (25.2)	235 (31.9)	227 (69.0)	334 (45.4)	< 0.001
HPV vaccination may improve the awareness of sexually transmitted diseases	122 (36.5)	193 (26.2)	185 (55.4)	407 (55.3)	18 (5.4)	103 (14.0)	9 (2.7)	20 (2.7)	< 0.001
HPV vaccination may reduce participation in cervical screening	3 (0.9)	37 (5.0)	86 (26.0)	310 (42.1)	135 (40.8)	236 (32.1)	107 (32.3)	138 (18.8)	< 0.001

^†^ Mann-Whitney U test.

### School Nurses’ Self-Perceived Knowledge About HPV Vaccination: A Comparison Between 2016 and 2024

Significant differences were found across all statements regarding self-perceived knowledge, with a higher proportion of school nurses in 2024 agreeing to having good knowledge about vaccine safety and to inform and respond to families’ questions, compared to 2016 (see Table 3). Still, almost half (45%) of all participants in 2024 reported they wished to receive more education about HPV.

**Table 3.** Self-Perceived Knowledge among Participating School Nurses in 2024 (n = 344) and 2016 (n = 736).

I have good knowledge about HPV:	Strongly agree n (%)		Partially Agree n (%)		Partially disagree n (%)		Strongly disagree n (%)		*p-value*†
	2024	2016	2024	2016	2024	2016	2024	2016	
to inform children before vaccination	281 (81.7)	454 (61.7)	63 (18.3)	214 (35.9)	0	7 (1.0)	0	0	< 0.001
to inform parents before vaccination	253 (73.8)	398 (54.1)	90 (26.2)	315 (42.8)	0	10 (1.4)	0	0	< 0.001
vaccines safety profile	192 (55.8)	284 (38.9)	147 (42.7)	419 (57.3)	5 (1.5)	27 (3.7)	0	1 (0.1)	< 0.001
to answer parents’ questions about HPV vaccine	202 (59.6)	292 (40.1)	132 (38.9)	415 (57.0)	4 (1.2)	20 (2.7)	1 (0.3)	1 (0.1)	< 0.001

* Question re-phrased due to introduction of the gender-neutral vaccination program. In 2016 “to inform girls”, in 2024 “to inform children”.

^†^ Mann-Whitney U test.

### School Nurses’ Experiences of Providing Information About HPV Vaccination

Results from the 2024 data regarding school nurses’ experiences of providing information about HPV vaccination are detailed in Table 4. The majority of participants provided written information about the vaccination to parents. More than half (56%) only reported to provide written information to parents, while 44% reported two or more formats, most frequently a combination of written, and telephone or e-mail on the initiative of the parents. About one third reported that they initiated contact by e-mail/telephone to provide information, and 17% provided information at teacher-parental meeting.

**Table 4.** School Nurses’ Experiences of Providing Information About HPV and HPV Vaccination in 2024 (n = 344*)*.

	n	(%)	Missing values
Parents have contacted me regarding HPV vaccination			
Strongly Agree	191	(56.0)	3
Partially agree	99	(29.0)	
Partially disagree	26	(7.6)	
Strongly disagree	25	(7.4)	
Children have asked me questions about the HPV vaccination			
Always	73	(21.6)	5
Usually	172	(50.7)	
Rarely	90	(26.5)	
Never	4	(1.2)	
My recommendations as a school nurse are important to the family's vaccination decision			
Strongly Agree	141	(42.0)	8
Partially agree	171	(50.9)	
Partially disagree	22	(6.5)	
Strongly disagree	2	(0.6)	
I contact parents who have declined or want to postpone HPV vaccination for further dialogue			
Always	126	(38.0)	12
Usually	92	(27.7)	
Rarely	79	(23.8)	
Never	35	(10.5)	
I offer a new vaccination opportunity to families who have declined HPV vaccination			7
Always	202	(59.9)	
Usually	64	(19.0)	
Rarely	43	(12.8)	
Never	28	(8.3)	
Whose responsibility is it that unvaccinated children are offered vaccination again?			
The parent's	107	(32.4)	14
The school nurse's	223	(67.6)	
The child's	0	0	
I inform children about the HPV vaccination orally in class			
Yes	273	(86.7)	29
No	42	(13.3)	
I inform parents about HPV vaccination through*:			
Written information	316	(91.9)	0
Parental meetings	58	(16.9)	
Telephone and/or e-mail initiated by parents	126	(36.6)	
Telephone and/or e-mail initiated by me	101	(29.4)	

* Multiple answers possible.

A majority of participants (85%) strongly or partially agreed that they had been contacted by parents about HPV vaccination and 92% strongly or partially agreed that their professional recommendations are important to families’ vaccination decision. Furthermore, approximately two-thirds of participants reported that they always or usually contacted parents who had declined or postponed vaccination. Regarding the responsibility for offering additional vaccination opportunities to unvaccinated children, 68% identified the school nurse as primarily responsible, while 32% considered it the parents’ responsibility.

The most reported reasons for parents to decline or postpone vaccination (multiple answers possible) was: fear of side effects (62%), the young age of the child (35%), the child's fear of needles (33%), religious reasons (24%), fear of sexual risk behavior (10%) and the child has other medical conditions (6%). Other reasons were reported by 21% while 6% stated they did not know. Other reasons included open-ended statements concerning: that parents had also declined other childhood vaccinations, lacked awareness regarding the importance of vaccinating boys and that parents of children with neurodevelopmental conditions often had more concerns about the need to vaccinate their child and to acknowledge that their child will become sexually active in the future.

Most school nurses (87%) provided children with oral information, in the classroom, prior to vaccination and 72% used the Public Health Agency's informational material. Furthermore, A similar proportion (72%) reported to always or usually receive questions from children about vaccination. The most frequently received questions from children (n = 320) regarded: concerns about fear of pain “will it hurt?” (55%), wanting to know more “what is it, why should I take it and what will happen if I don’t?” (37%), “are there any side-effects?” (4%), about the vaccination procedure “what will happen?” (4%), “why do boys get vaccinated” (4%),”do I have to get the shot?” (4%), “why do I have to get two doses?” (3%), questions about sexual transmission (2%) and “I wish to get vaccinated but my parents do not consent, what do I do?” (1%).

### School Nurses’ Experiences of Barriers and Facilitators to HPV Vaccination: Applying the COM-B Model

Data from three open-ended questions in the 2024 questionnaire were used for the thematic analysis: “Which strategies have you used when communicating with hesitant parents?” (n = 304), “I have experienced the following difficulties with HPV vaccinations” (n = 279) and “Additional comments about HPV vaccinations” (n = 132). The results of the qualitative analysis are presented in detail in Table 5, accompanied by quotes from the original data. The analysis identified barriers and facilitators for five different behaviors related to school nurses’ work with HPV: Informing children and parents about the vaccination, motivating hesitant parents, ensuring children's participation in healthcare decisions, managing children's fear of needles, and providing additional vaccination opportunities. For each behavior, sub-themes of barriers and facilitators are presented according to the main themes: Capability, Opportunity, and Motivation. The results are summarized below under each identified behavior.

**Table 5.** Results from the Thematic Analysis of Open-Ended Questions to Swedish School Nurses (2024), Applying the COM-B Model.

COM-B Theme	Sub-themes of barriers and facilitators	Quotation
	Informing Children and Parents About the Vaccination	
Capability	Ability to utilize appropriate information strategies and materials	*“I participate in the parental meeting and show the PHAs film about HPV that I think is very good, later I show this film in the classroom” (#388)*
Opportunity	Availability of information material and views of professional responsibility	*“Many are struggling with the Swedish language. Then the information is difficult to understand” (#188)*
Opportunity	Time-consuming to comply with informed consent practices	*“The most difficult thing is to get approval from all guardians. This is incredibly time-consuming but I choose to give it much time” (#123)*
Motivation	Experiences of HPV-related disease	*“I have worked 16 years with sexually transmitted diseases and seen the consequences of condyloma” (#447)*
	Motivating Hesitant Parents	
Capability	Ability and confidence to address concerns of parents and children	*“It's hard to know when to call or not… I would have wished for more education about how to address those who decline” (#206)*
Capability	Ability to build parental trust and apply cultural awareness and sensitivity	*“When I told them about my professional confidentiality some have consented to vaccination since they are scared about what other parents with the same religion, culture or country of origin will say or think” (#293)*
Opportunity	Restricted time for parental dialogue and lack of uniform guidelines to manage vaccine hesitancy	*“It's difficult to find time to prioritize conversations with sceptic parents since I as a school nurse have so many other parallel tasks…these conversations take time.” (#433)*
Opportunity	Limited social influence to counter public distrust and a need for awareness campaigns	*“A country-wide campaign to address ‘the anti-vaccine-movement’ would be needed. It's still not a very large group but I feel that resistance is growing.” (#422)*
Opportunity	Need for education and training to communicate with hesitant parents	*“I would like to receive more education about how to address those who decline” (#207)*
Motivation	Professional pride, seeing results and views of professional responsibility	*“I don’t see it as my job to influence parents in their decision” (#282)*
	Ensuring Children's Participation in Healthcare Decisions	
Opportunity	Lacking uniform guidelines about children's opportunity to self-consent	*“[Challenging when] The child wishes to self-consent, now we solve it by vaccinating after they have turned 18 years of age (#80)*
Motivation	Views of children's ability and maturity to participate in decision-making	*“I just talk, I don’t believe it's the children themselves who needs knowledge about the vaccine, they are not making the decision” (#113)*
	Managing Children's Fear of Needles	
Capability	Using strategies to handle children's fear and avoid worry and stress spreading among peers	*“…we have let the pupils play Minecraft virus and vaccine for them to be involved and learn about why vaccines are important. They have also been shown the injection needles and had the opportunity to give dolls the shot in order to reduce fear of needles and pain” (#289)*
Opportunity	Utilize interdisciplinary collaboration to overcome fear of needles	*“…In some cases, I refer them for cognitive behavioral therapy, but it's difficult to find forums for children with severe fear.” (#129)*
Opportunity	Time-consuming to support children with fear of needles	*“[support is] Heavy work that requires a lot of us as school nurses in the form om motivating and comforting children, and its time-consuming” (#119)*
	Providing Additional Vaccination Opportunities	
Opportunity	Lack of uniform work methods and restricted time	*“It's difficult with the number of pupils that I have to try to provide a new chance to consent during fifth grade” (#267)*
Motivation	View of professional role versus parental autonomy and responsibility	*“My opinion is that if parents decline when the vaccination is offered in fifth grade, then it's up to them if/when they change their mind.” (#433)*

#### Informing Children and Parents About the HPV Virus and Vaccination

Participants described that facilitators to successfully promoting HPV vaccination were the ability to utilize appropriate information strategies and materials. To provide information in several formats was considered a means to adapt information to the individual needs of parents and children. A common strategy was to provide information repeatedly. Attending teacher-parental meetings were described as a good forum to provide a combination of face-to-face information followed by written information. Some described that they informed about the vaccination already at parental meetings in the child's first school year.

Informing children in class was regarded by participants as both a facilitator of children's participation and an opportunity for them to favorably influence the parents. The content of the information participants provided were influenced by their own views of children's maturity. Beliefs that children were too young to comprehend information or to participate in decision-making discouraged the nurses from providing extensive information. Instead, they focused more on the practicalities. Participants views about professional responsibilities to promote vaccinations influenced their strategies. It was argued that written information was sufficient and it was a parental responsibility to seek more knowledge. A practical example was to delegate the provision of vaccination information, both to children and parents, to the teachers.

Participants found it challenging to provide information to parents and children who were not fluent in Swedish or had low health literacy. Efforts to make sure the information was given in an appropriate language were prioritized but not always possible. They voiced a need for material adapted to different languages and different health literacy levels, and more clearly explained why boys should be vaccinated. A few stated that they used interpreters when communicating with parents who spoke a different first language.

To have personal experience of HPV-related disease and seeing the effects first-hand strengthened the sense of importance and opportunity to prevent disease. On the other hand, it could also make it difficult to talk about the subject and give a sense of hopelessness when knowing that some children would remain unprotected.

To comply with informed consent practices were seen as very important, but also described as the most tedious and time-consuming barrier. The school nurses prioritized most of their time to ensure that the formal documentation was correctly received in time. Furthermore, consent practices were described as rigid and not adapted to the complexity of different family situations such as when the school nurse cannot reach the parents and consequently not vaccinate.

#### Motivating Hesitant Parents

Participants described a lack of knowledge, training and confidence to address parental concerns and reasons for vaccine hesitancy. They also voiced an uncertainty about their professional mandate to contact hesitant parents, and the lack of guidelines to do so*.* Believing it was a parental responsibility to initiate dialogue with the school nurse, or not wanting to interfere in parental decisions discouraged active outreach. Further, a commonly expressed barrier was a lack of time.

On the other hand, nurses stated that they were motivated to reach out to hesitant parents because they had reflected about family circumstances that may call for additional support. Other motivational factors to actively motivate hesitant parents were professional pride, to take part in the elimination of cervical cancer, and seeing results of increased vaccine acceptance. Furthermore, cultural awareness and sensitivity were described as facilitators for addressing socio-cultural reasons for hesitancy. To be able to build parental trust facilitated not only vaccination dialogues but also fostered confidence in all health encounters with the school nurse. Multiple interactions and respectful dialogue with parents, during their child's schooling, facilitated fruitful relationships. Therefore, they voiced the importance of having present and reliable school health services where school nurses have time for the pupils and their parents.

The nurses described having limited social influence to counter public mistrust against vaccinations. Knowing that some families decline all vaccines, as well as experiences of parents rejecting further dialogue with the school nurse, were discouraging to continued efforts. Instead, participants voiced a need for awareness campaigns about the HPV vaccine and the introduction of a gender-neutral vaccination program.

#### Ensuring Children's Participation in Healthcare Decisions

The experience of conflicting wishes about vaccination within a family, either between the parents and the child or between parents, was described as a dilemma. Participants felt uncertain how to handle such situations. If the child wanted to get vaccinated but one or both parents decided against, nurses lacked confidence about letting the child's wish be final. Participants described that they informed the child about the possibility to get vaccinated later on, at an older age when they could self-consent. However, nurses described an uncertainty about how and when to conduct a maturity evaluation in order for the child to legally be allowed to self-consent, especially at younger ages. Furthermore, differing recommendations or guidelines concerning at what age children could self-consent was described. Statements about the recommendations and guidelines nurses complied with in this matter widely varied. Several stated that children had to wait until their 18^th^ birthday.

#### Managing Children's Fear of Needles

The ability to counter spread of worry and stress among children was described to be important. That children talked among each other about the vaccine being painful or shared misinformation about its safety could increase the fear of being vaccinated. This behavior among children was described to be frequent among children aged 11 to 12, but common in all age groups. The fear of pain was also described to affect acceptance of the second dose. Parents of children with a pre-existing fear of needles were perceived to more easily de-prioritize the HPV vaccination. Furthermore, experiences of children declining due to fear of needles when permitted to choose for themselves were described. To properly inform and communicate with children, teachers, and caregivers facilitated a calm environment.

Knowledge about the importance of timely vaccinations was a motivational factor to prioritize giving support to children with fear of needles. The ability to use different strategies was then an important facilitator. Examples given were using pain relieving plasters, diversion, relaxation techniques and having parents present*.* However, this could be time-consuming. Therefore, interdisciplinary collaboration was seen as important. The opportunity to refer to other medical professions either within or outside the school health services, was also a facilitating factor. For example, children had been referred to cognitive behavior therapy, hospital play therapy or medical interventions such as nitro oxygen during vaccination.

#### Providing Additional Vaccination Opportunities

Different approaches to provide unvaccinated children with an additional vaccination offer were described. Local methods included providing new offers and information at start of every new school year, as a set talking point at the repeated mandatory health talks provided by the school health services. The nurses’ own view of their professional responsibilities motivated initiatives to actively provide additional vaccination offers. The various statements illustrated a lack of uniform methods, and restricted time was again voiced as a barrier.

## Discussion

### Main Findings

In general, more school nurses reported favorable attitudes towards, and good self-perceived knowledge about, HPV and HPV vaccinations, comparing results from 2024 and 2016. Most agreed to have been contacted by parents with concerns about the vaccination about the vaccination (85%), and to have received questions from children (72%). More than half (56%) of participants only reported to provide parents with written information about the vaccination. Furthermore, the majority (92%) agreed that their recommendation was important to families’ vaccination decision. However, 21% rarely or never offered a new opportunity to families who had initially declined vaccination and almost a third considered it to be the parents’ responsibility that unvaccinated children are offered a new opportunity. The thematic analysis of open-ended data identified several important barriers and facilitators connected to the COM-B themes of capability, opportunity and motivation to behaviors related to HPV vaccination in the school-based setting. These findings can inform future intervention development with the aim to strengthen school nurses’ important work.

#### Capability

One explanation for HPV vaccination rates being lower compared to other childhood vaccinations in Sweden is the transition from vaccinations being provided at child health clinics to the school health services (Appelqvist et al., 2023). In the ages 0–5 parents regularly accompany their children to health centers for check-ups and vaccinations where multiple interactions with a nurse overtime facilitates a trusting relationship (Appelqvist et al., 2023). The school health services, on the other hand, are predominantly communicating with parents through written information, as also seen in this study. To inform and promote the vaccine at the teacher- parental meetings was one way of bridging this gap. Unfortunately, only a minority of school nurses used this strategy. Furthermore, language and health literacy barriers are likely to be more prominent when information is provided mainly in written form (Grandahl et al., 2015; Sanders et al., 2009). Although school-based vaccination programs are regarded as the most equitable approach to delivering HPV vaccines, Swedish girls whose parents have lower income, lower educational attainment, or an immigrant background are less likely to receive the HPV vaccine (Wang et al., 2019; Wemrell et al., 2023), highlighting the need for more inclusive communication strategies and targeted outreach.

#### Opportunity

HCPs’ confidence about the benefits and safety of vaccinations is known to affect their recommendations to families (Garbutt et al., 2023; Karlsson et al., 2019; Lin et al., 2021). Although the majority of participants in 2024 reported good self-perceived knowledge about the vaccine and to be able to inform families, 45% still expressed a desire for further education. Specifically, they voiced a need for more knowledge and training about how to address vaccine hesitancy, especially to counter religious and cultural concerns among parents, which is in line with previous studies (Enskär et al., 2023; Runngren et al., 2022). Furthermore, there is a need to increase evidence-based knowledge and eradicate common misconceptions, foremost of which is the idea that vaccination will lead to increased sexual risk behavior (Grandahl et al., 2017). Sadly, these misconceptions were also present among school nurses in this study.

The participants described that parents of children with neuropsychiatric disabilities had more questions and concerns. A recent Swedish nationwide registry-based study found a lower vaccination coverage among girls with mental illness and neurodevelopmental conditions (Hu et al., 2024). These findings call for further research to explore underlying barriers to vaccine acceptance among these children and their parents. This is especially important since Swedish women with severe mental illness participate less in cervical screening and have a doubled risk of invasive cervical cancer (Hu et al., 2023). Furthermore, HCPs may benefit from education about the effectiveness of the vaccine, the lack of serious side effects, the importance of timely vaccinations and the importance of also vaccinating boys (Grandahl & Nevéus, 2021).

Fear of needles is a barrier for vaccine acceptance both from the perspective of HCPs and children. The ability to prepare children and use strategies to manage children's fear facilitated vaccine acceptance, while a barrier was that this was time-consuming. School nurses involved in HPV vaccination should be enabled to address children's individual need of support rather than to optimize workflow (Lindén & Odenbring, 2022).

To increase awareness about legal aspects of consent practices and children's right to participation in health decisions may also support school nurses to ensure children's participation. It's important to note that children's participation is not identical to self-consent but involves the right for the child to be informed and to speak their mind. However, lack of awareness about the legal aspects of children's right to self-consent and about how to conduct a maturity evaluation of the child has previously been reported as possible barriers for children's participation and uptake of HPV vaccinations (Audrey et al., 2020; Fisher et al., 2019; Karafillakis et al., 2021; Zimet et al., 2021). It's crucial that HCPs are aware and confident about adolescents’ legal right to self-consent and for them to communicate this to adolescents (McNally et al., 2023; Zimet et al., 2021). It is noteworthy that the school nurses participating in the present study emphasized the importance of building trust with families. From their perspective, this also presents a challenge, as allowing children to self-consent may risk undermining parental trust. Our results indicate a need for medical law education and uniform legal interpretations, at policy level, for school nurses to adhere to.

#### Motivation

In line with previous studies, we found that school nurses’ views of their professional responsibility affected their work methods in terms of active outreach to hesitant parents and provision of providing additional offers to unvaccinated children (Enskär et al., 2023; McNally et al., 2023). To empower school nurses by emphasizing their key role for the success of the HPV vaccination program and the importance of their strong recommendations for parental decision-making may strengthen their professional pride. The need of public awareness campaigns has previously been raised and could strengthen their role (Dionne et al., 2023). In addition, they need a clarified mandate for vaccine promotion and reliable routines for when and how to contact hesitant families.

### Strengths and Limitations

The sample in the current study was similar to the 2016 study, however the sample size was smaller compared to the 2016 study despite similar recruitment strategies. One possible explanation is the impact of the COVID-19 pandemic, which may have disrupted the daily work routines of school nurses. Currently, there is no available data specifying how many employed Swedish school nurses meet the eligibility criteria—namely, those involved in administering HPV vaccinations. Although HPV vaccination is primarily offered in fifth grade, unvaccinated children can receive the vaccine through school health services until the age of 18. Moreover, high staff turnover and regional variations in the organization of school health services further complicate efforts to estimate the total number of eligible school nurses. After consultation with a statistician, it was concluded that a formal pre-study sample size calculation was not feasible. Nevertheless, the sample was considered adequate due to its broad representation of school nurses from all 21 regions in Sweden.

As described, modifications have been made to the questionnaire since 2016, which limits comparability. However, given that NIPs and school health services evolve over time, it is important to adapt the questionnaire to reflect current practices. Additionally, the analysis of both quantitative and qualitative data provides deeper insight into barriers and facilitators at both individual and organizational levels.

### Implications for School Nursing

Although HPV vaccine coverage is high in Sweden, continued efforts are necessary to ensure all children are protected against future HPV-related cancers. School nurses play a key role in the delivery of the HPV vaccination program, yet vaccination is a complex task and only one of many responsibilities they manage. The participating nurses reported good self-perceived knowledge and positive attitudes toward the HPV vaccine. However, they also expressed a clear need for further education and training, particularly in addressing vaccine hesitancy among parents. Additionally, lack of tailored tools and resources, such as culturally and linguistically appropriate informational materials and sufficient time allocated for vaccination-related tasks, limits their capacity to effectively promote, administer, and follow up on HPV vaccinations. Consistent with previous research (Enskär et al., 2023; Enskär et al., 2024; Runngren et al., 2022) our results show a variation in work methods, legal interpretations, views of professional responsibilities, and guidelines which suggest a lack of uniformity in provision of HPV vaccinations in the school-based program. This is worrying since it may affect the equity of the NIP. In alignment with the Immunization Agenda 2030 - ensuring no child is left behind -, and to achieve the goal of eliminating cervical cancer, further efforts to strengthen school health services are essential to promote equity in immunization programs (World Health Organization, 2020b). Based on our findings, we propose the following actions to strengthen the school-based HPV vaccination program and the school nurses key role for HPV vaccinations

- employ uniform guidelines to improve equity in the immunization program.
- develop tailored informational tools and increase awareness of existing resources among school nurses.
- provide ongoing education and training on children's rights to participate in healthcare decisions, strategies to address vaccine hesitancy, techniques for managing needle fear, and ways to counteract common misconceptions about HPV vaccination – including among healthcare professionals.
- launch awareness campaigns emphasizing the importance of timely, gender-neutral vaccination and highlighting the school nurse's role as a trusted source of information.

## Supplemental Material

sj-pdf-1-jsn-10.1177_10598405251374726 - Supplemental material for School Nurses’ Experiences of the School-Based HPV Vaccination Program – A Decade Later Supplemental material, sj-pdf-1-jsn-10.1177_10598405251374726 for School Nurses’ Experiences of the School-Based HPV Vaccination Program – A Decade Later by Ida Enskär, Tryggve Nevéus, Karin Enskär, Carolina Källqvist and Maria Grandahl in The Journal of School Nursing

## References

[bibr1-10598405251374726] Appelqvist E. Danielsson M. Jama A. Ask L. S. Stenhammar C. Lindstrand A. Riesbeck K. Roth A. (2023). Parental views and the key role of nurses for high vaccine acceptance in Sweden - a focus group study. BMC Public Health, 23(1), 1786. 10.1186/s12889-023-16678-5 PMC10500778 37710197

[bibr2-10598405251374726] Audrey S. Farr M. Roderick M. Evans K. Fisher H. (2020). How acceptable is adolescent self-consent for the HPV vaccination: Findings from a qualitative study in south-west England. Vaccine, 38(47), 7472–7478. 10.1016/j.vaccine.2020.09.074 PMC7604563 33041101

[bibr3-10598405251374726] Braun V. Clarke V. (2006). Using thematic analysis in psychology. Qualitative Research in Psychology, 3(2), 77–101. 10.1191/1478088706qp063oa

[bibr4-10598405251374726] Casey R. M. Akaba H. Hyde T. B. Bloem P. (2024). COVID-19 pandemic and equity of global human papillomavirus vaccination: Descriptive study of world health organization-unicef vaccination coverage estimates. BMJ Medicine, 3(1), e000726. 10.1136/bmjmed-2023-000726 PMC10826539 38293682

[bibr5-10598405251374726] Dionne M. Sauvageau C. Kiely M. Rathwell M. Bandara T. Neudorf C. Dubé È (2023). The problem is not lack of information": A qualitative study of parents and school nurses’ perceptions of barriers and potential solutions for HPV vaccination in schools. Vaccine, 41(45), 6654–6660. 10.1016/j.vaccine.2023.09.054 37777452

[bibr6-10598405251374726] Enskär I. Enskär K. Nevéus T. Hess Engström A. Grandahl M. (2023). Barriers in the school-based pan-gender HPV vaccination program in Sweden: Healthcare providers perspectives. Vaccines, 11(2), 310. 10.3390/vaccines11020310 PMC9962905 36851188

[bibr7-10598405251374726] Enskär I. Fransson E. Enskär K. Nevéus T. Grandahl M. (2024). School children's perceptions about being offered the HPV vaccination - A focus group study. Acta Paediatrica, 113(7), 1672–1678. 10.1111/apa.17225 38551330

[bibr8-10598405251374726] Fisher H. Harding S. Hickman M. Macleod J. Audrey S. (2019). Barriers and enablers to adolescent self-consent for vaccination: A mixed-methods evidence synthesis. Vaccine, 37(3), 417–429. 10.1016/j.vaccine.2018.12.007 PMC6453814 30573357

[bibr9-10598405251374726] Garbutt J. Wang R. Graham S. McKay V. Haire-Joshu D. Barker A. Liu L. (2023). Provider and practice factors associated with on-time HPV vaccination in primary care. Academic Pediatrics, 23(4), 800–807. 10.1016/j.acap.2022.12.015 36592791

[bibr10-10598405251374726] Grandahl M. Larsson M. Tyden T. Stenhammar C. (2017). School nurses’ attitudes towards and experiences of the Swedish school-based HPV vaccination programme - A repeated cross sectional study. PLoS One, 12(4), e0175883. 10.1371/journal.pone.0175883 PMC5395208 28419156

[bibr11-10598405251374726] Grandahl M. Neveus T. Dalianis T. Larsson M. Tyden T. Stenhammar C. (2019). I also want to be vaccinated!’ - adolescent boys’ awareness and thoughts, perceived benefits, information sources, and intention to be vaccinated against human papillomavirus (HPV). Human Vaccine & Immunotherapeutics, 15(7-8), 1794–1802. 10.1080/21645515.2018.1551670 PMC6746528 30481108

[bibr12-10598405251374726] Grandahl M. Nevéus T. (2021). Barriers towards HPV vaccinations for boys and young men: A narrative review. Viruses, 13(8), 1644. 10.3390/v13081644 PMC8402923 34452508

[bibr13-10598405251374726] Grandahl M. Tydén T. Gottvall M. Westerling R. Oscarsson M. (2015). Immigrant women's experiences and views on the prevention of cervical cancer: A qualitative study. Health Expectations, 18(3), 344–354. 10.1111/hex.12034 PMC5060783 23252449

[bibr14-10598405251374726] Grandahl M. Tydén T. Rosenblad A. Oscarsson M. Nevéus T. Stenhammar C. (2014). School nurses’ attitudes and experiences regarding the human papillomavirus vaccination programme in Sweden: A population-based survey. BMC Public Health, 14, 540. 10.1186/1471-2458-14-540 PMC4061918 24886332

[bibr15-10598405251374726] Hanley S. J. B. Yoshioka E. Ito Y. Kishi R. (2015). HPV Vaccination crisis in Japan. The Lancet, 385(9987), 2571. 10.1016/S0140-6736(15)61152-7 26122153

[bibr16-10598405251374726] Harris P. A. Taylor R. Minor B. L. Elliott V. Fernandez M. O'Neal L. McLeod L. Delacqua G. Delacqua F. Kirby J. Duda S. N. (2019). The REDCap consortium: Building an international community of software platform partners. Journal of Biomedical Informatics, 95, 103208. 10.1016/j.jbi.2019.103208 PMC7254481 31078660

[bibr17-10598405251374726] Harris P. A. Taylor R. Thielke R. Payne J. Gonzalez N. Conde J. G. (2009). Research electronic data capture (REDCap)—A metadata-driven methodology and workflow process for providing translational research informatics support. Journal of Biomedical Informatics, 42(2), 377–381. 10.1016/j.jbi.2008.08.010 PMC2700030 18929686

[bibr18-10598405251374726] Hu K. Barker M. M. Herweijer E. Wang J. Feldman A. L. Lu D. Valdimarsdóttir U. Sundström K. Fang F. (2024). The role of mental illness and neurodevelopmental conditions in human papillomavirus vaccination uptake within the Swedish school-based vaccination programme: A population-based cohort study. The Lancet Public Health, 9(9), e674–e683. 10.1016/S2468-2667(24)00182-8 39214636

[bibr19-10598405251374726] Hu K. Wang J. Sparén P. Herweijer E. Sjölander A. Adami H.-O. Valdimarsdóttir U. Sundström K. Fang F. (2023). Invasive cervical cancer, precancerous lesions, and cervical screening participation among women with mental illness in Sweden: A population-based observational study. The Lancet Public Health, 8(4), e266–e275. 10.1016/S2468-2667(23)00026-9 36965981

[bibr20-10598405251374726] Karafillakis E. Peretti-Watel P. Verger P. Chantler T. Larson H. J. (2021). The role of maturity in adolescent decision-making around HPV vaccination in France. Vaccine, 39(40), 5741–5747. 10.1016/j.vaccine.2021.08.096 34483023

[bibr21-10598405251374726] Karafillakis E. Van Damme P. Hendrickx G. Larson H. J. (2022). COVID-19 in Europe: New challenges for addressing vaccine hesitancy. The Lancet, 399(10326), 699–701. 10.1016/S0140-6736(22)00150-7 PMC8813064 35123665

[bibr22-10598405251374726] Karlsson L. C. Lewandowsky S. Antfolk J. Salo P. Lindfelt M. Oksanen T. Kivimäki M. Soveri A. (2019). The association between vaccination confidence, vaccination behavior, and willingness to recommend vaccines among Finnish healthcare workers. PLoS One, 14(10), e0224330. 10.1371/journal.pone.0224330 PMC6822763 31671115

[bibr23-10598405251374726] Lin C. Mullen J. Smith D. Kotarba M. Kaplan S. J. Tu P. (2021). Healthcare Providers’ vaccine perceptions, hesitancy, and recommendation to patients: A systematic review. Vaccines, 9(7), 713. 10.3390/vaccines9070713 PMC8310254 34358132

[bibr24-10598405251374726] Lindén L. Odenbring Y. (2022). The needle pricking and two modes of ‘doing good’ in the Swedish school-based human papillomavirus vaccination programme. Sociology of Health & Illness, 44(8), 1344–1360. 10.1111/1467-9566.13515 PMC9545275 35932249

[bibr25-10598405251374726] López N. Garcés-Sánchez M. Panizo M. B. de la Cueva I. S. Artés M. T. Ramos B. Cotarelo M. (2020). HPV Knowledge and vaccine acceptance among European adolescents and their parents: A systematic literature review. Public Health Reviews, 41(1), 10. 10.1186/s40985-020-00126-5 PMC7222509 32435520

[bibr26-10598405251374726] MacDonald N. E. (2015). Vaccine hesitancy: Definition, scope and determinants. Vaccine, 33(34), 4161–4164. 10.1016/j.vaccine.2015.04.036 25896383

[bibr27-10598405251374726] McNally K. Roess A. Weinstein A. Lindley L. Wallin R. (2023). School Nurses’ experiences and roles in promoting and administering the HPV vaccine: A systematic review using the socioecological framework. The Journal of School Nursing, 40(1), 43–57. 10.1177/10598405231206109 37828750

[bibr28-10598405251374726] Michie S. van Stralen M. M. West R. (2011). The behaviour change wheel: A new method for characterising and designing behaviour change interventions. Implementation Science, 6(1), 42. 10.1186/1748-5908-6-42 PMC3096582 21513547

[bibr29-10598405251374726] The Public Health Agency of Sweden (PHA) . (2022). Lägesrapport om de nationella vaccinationsprogrammen 2022. Folkhälsomyndigheten. https://www.folkhalsomyndigheten.se/publikationer-och-material/publikationsarkiv/l/lagesrapport-om-de-nationella-vaccinationsprogrammen-2022/.

[bibr30-10598405251374726] The Public Health Agency of Sweden (PHA) . (2023). Vaccinationstäckning för vaccinationer givna inom elevhälsan. https://www.folkhalsomyndigheten.se/faktablad/barnvaccinationer/.

[bibr31-10598405251374726] Riksföreningen för skolsköterskor . (n.d.). Riksföreningen för Skolsköterskor. En ideell professionsförening för dig som skolsköterska. Riksföreningen för skolsköterskor. https://swenurse.se/sektionerochnatverk/riksforeningenforskolskoterskor.

[bibr32-10598405251374726] Runngren E. Eriksson M. Blomberg K. (2022). Balancing between being proactive and neutral: School Nurses’ experiences of offering human papilloma virus vaccination to girls. Journal of School Nursing, 38(3), 270–278. 10.1177/1059840520933323 PMC9069651 32578487

[bibr33-10598405251374726] Sanders L. M. Federico S. Klass P. Abrams M. A. Dreyer B. (2009). Literacy and child health: A systematic review. Archives of Pediatrics & Adolescent Medicine, 163(2), 131–140. 10.1001/archpediatrics.2008.539 19188645

[bibr34-10598405251374726] Shapiro G. K. (2022). HPV Vaccination: An underused strategy for the prevention of cancer. Current Oncology, 29(5), 3780–3792. https://www.mdpi.com/1718-7729/29/5/303. https://doi.org/10.3390/curroncol29050303 10.3390/curroncol29050303 PMC9140027 35621693

[bibr35-10598405251374726] Sung H. Ferlay J. Siegel R. L. Laversanne M. Soerjomataram I. Jemal A. Bray F. (2021). Global cancer statistics 2020: GLOBOCAN estimates of incidence and mortality worldwide for 36 cancers in 185 countries. CA Cancer Journal for Clinicians, 71(3), 209–249. 10.3322/caac.21660 33538338

[bibr36-10598405251374726] Suppli C. H. Hansen N. D. Rasmussen M. Valentiner-Branth P. Krause T. G. Molbak K. (2018). Decline in HPV-vaccination uptake in Denmark - the association between HPV-related media coverage and HPV-vaccination. BMC Public Health, 18(1), 1360. 10.1186/s12889-018-6268-x PMC6288961 30526589

[bibr37-10598405251374726] The Swedish Association of Local Authorities and Regions . (2025). Elevhälsa, statistik för personalgrupper. https://skr.se/skr/skolakulturfritid/forskolagrundochgymnasieskolakomvux/elevhalsaskolmat/elevhalsa/statistikpersonalgrupper.70066.html.

[bibr38-10598405251374726] Wang J. Ploner A. Sparén P. Lepp T. Roth A. Arnheim-Dahlström L. Sundström K. (2019). Mode of HPV vaccination delivery and equity in vaccine uptake: A nationwide cohort study. Preventive Medicine, 120, 26–33. 10.1016/j.ypmed.2018.12.014 30593796

[bibr39-10598405251374726] Wemrell M. Vicente R. P. Merlo J. (2023). Mapping sociodemographic and geographical differences in human papillomavirus non-vaccination among young girls in Sweden. Scandinavian Journal of Public Health, 51(2), 288–295. 10.1177/14034948221075410 PMC9969304 35120427

[bibr40-10598405251374726] World Health Organization (WHO) . (2019a). Ten threats to global health in 2019. Retrieved from https://www.who.int/news-room/spotlight/ten-threats-to-global-health-in-2019.

[bibr41-10598405251374726] World Health Organization (WHO) . (2019b). TIP Tailoring Immunization Programmes. https://www.who.int/europe/publications/i/item/9789289054492.

[bibr42-10598405251374726] World Health Organization (WHO) . (2020a). Global strategy to accelerate the elimination of cervical cancer as a public health problem. Retrieved from https://www.who.int/publications/i/item/9789240014107.

[bibr43-10598405251374726] World Health Organization (WHO) . (2020b). Immunization Agenda 2030: A Global Strategy to Leave No One Behind. Retrieved from https://www.who.int/teams/immunization-vaccines-and-biologicals/strategies/ia2030.

[bibr44-10598405251374726] Zimet G. D. Silverman R. D. Bednarczyk R. A. English A. (2021). Adolescent consent for human papillomavirus vaccine: Ethical, legal, and practical considerations. The Journal of Pediatrics, 231, 24–30. 10.1016/j.jpeds.2021.01.026 PMC8005441 33484694

