# Supplementary material for: School Nurses’ Experiences of the School-Based HPV Vaccination Program – A Decade Later
Source: J Sch Nurs. 2025 Sep 8;42(6):581–94. doi: 10.1177/10598405251374726 (PMC13727988; doi:10.1177/10598405251374726)
Supplement: Supplementary file 1 [file sj-pdf-1-jsn-10.1177_10598405251374726.pdf]

UPPSALA
UNIVERSITET

To School nurses vaccinating against HPV in the school-based vaccination program in Sweden 2023-2024

1. I have good knowledge about HPV to inform children before the vaccination
   - ☐ strongly agree
   - ☐ partially agree
   - ☐ partially disagree
   - ☐ strongly disagree

2. I have good knowledge about HPV to inform parents (legal guardians) before the vaccination
   - ☐ strongly agree
   - ☐ partially agree
   - ☐ partially disagree
   - ☐ strongly disagree

3. I have good knowledge about the safety profile of the HPV-vaccines (i.e side effects)
   - ☐ strongly agree
   - ☐ partially agree
   - ☐ partially disagree
   - ☐ strongly disagree

4. I wish to receive more education about the HPV virus and vaccine
   - ☐ Yes
   - ☐ No

5. Parents (legal guardians) have contacted me as a school nurse about HPV vaccination
   - ☐ strongly agree
   - ☐ partially agree
   - ☐ partially disagree
   - ☐ strongly disagree

6. The most common question I receive from parents (legal guardians) about HPV vaccination is

   ____

7. When the parents (legal guardians) asks questions about HPV vaccine, I have good knowledge to answer the questions
   - ☐ strongly agree
   - ☐ partially agree
   - ☐ partially disagree
   - ☐ strongly disagree

©Copy rights reserved Maria Grandahl, Uppsala University. Please cite Grandahl et al. when using the questionnaire.

UPPSALA
UNIVERSITET

8. Children have asked me questions about the HPV vaccination
   - ☐ Always
   - ☐ Usually
   - ☐ Rarely
   - ☐ Never

9. The most frequently received question about HPV vaccination I receive from children is____

10. I inform the students orally before the vaccination (additional options may be possible)
   - ☐ I inform orally in class
   - ☐ I do not provide oral information

11. I use the following informational material when informing children about HPV vaccinations
   - ☐ the public health agencies informational material on HPV vaccination to children
   - ☐ other:____

12. I inform the parents (legal guardians) about HPV vaccination in/by
(additional options may be possible)
   - ☐ Written information
   - ☐ Information at parental meetings
   - ☐ Telephone/email-/ contact by e mail on the parents' initiative
   - ☐ Telephone/email-/ contact by e mail on my initiative

   Comments :__________________________

13. I agree that it was appropriate that HPV vaccinations were introduced into the general childhood vaccination programme
   - ☐ strongly agree
   - ☐ partially agree
   - ☐ partially disagree
   - ☐ strongly disagree

14. Below are some statements about HPV vaccination. Please mark the most suitable answer

©Copy rights reserved Maria Grandahl, Uppsala University. Please cite Grandahl et al. when using the questionnaire.

UPPSALA
UNIVERSITET

		Strongly agree	Partially agree	Partially disagree	Strongly disagree
**a)**	The HPV vaccine can cause serious adverse effects	☐	☐	☐	☐
**b)**	HPV vaccination is more painful than other vaccinations	☐	☐	☐	☐
**c)**	Fear of needles is a problem for HPV vaccination	☐	☐	☐	☐
**d)**	Those who decline vaccination should be offered the vaccination later	☐	☐	☐	☐
**e)**	I have sufficient confidence in the authorities' decisions about HPV vaccination	☐	☐	☐	☐
**f)**	HPV vaccination may result in decreased condom use	☐	☐	☐	☐
**g)**	HPV-vaccination may result in increased number of sexual partners.	☐	☐	☐	☐
**h)**	HPV-vaccination may increase awareness of sexually transmitted infections	☐	☐	☐	☐
**i)**	HPV vaccination can lead to decreased attendance in cervical cancer screening programme	☐	☐	☐	☐

15. The coverage for HPV vaccinations at my school has been
- ☐ Less than or equal to 70 %
- ☐ 71-80 %
- ☐ 81-90 %
- ☐ 91-100 %
- ☐ Do not know

©Copy rights reserved Maria Grandahl, Uppsala University. Please cite Grandahl et al. when using the questionnaire.

UPPSALA
UNIVERSITET

16. I believe the most common reason for parents not consenting to or to postpone HPV vaccination is

- ☐ the child is too young
- ☐ fear of side effects
- ☐ religious reasons
- ☐ fear of sexual risk behaviour
- ☐ the child has another ongoing disease
- ☐ do not know
- ☐ other reason, please specify

17. I contact parents who have declined vaccination for further dialogue
- ☐ Always
- ☐ Usually
- ☐ Rarely
- ☐ Never

18. I offer a new vaccination opportunity to families who have declined vaccination
- ☐ Always
- ☐ Usually
- ☐ Rarely
- ☐ Never

19. Whose responsibility is it that the child is offered a new opportunity to get vaccinated?
- ☐ The school nurses
- ☐ The parents
- ☐ The child

20. My recommendations are important for families' vaccination-decisions
- ☐ strongly agree
- ☐ partially agree
- ☐ partially disagree
- ☐ strongly disagree

21. Which strategies have you used use when communicating with hesitant parents?:

If any were the strategies successful?

©Copy rights reserved Maria Grandahl, Uppsala University. Please cite Grandahl et al. when using the questionnaire.

UPPSALA
UNIVERSITET

22. I have experienced the following difficulties with the HPV vaccinations

23. Additional comments about HPV vaccinations

24. I am born in 19

25. I am

- ☐ female
- ☐ male
- ☐ other
- ☐ Do not want to answer

26. If you are a parent (legal guardian), are your child/children vaccinated according to the national immunization program?
- ☐ Yes
- ☐ No
- ☐ Not applicable

27. Have you been vaccinated against COVID-19?
- ☐ Yes
- ☐ No
- ☐ I do not want to answer

28. I work at a
- ☐ public school
- ☐ private school

29. I work in a school located (if you work in more than one school additional options are possible)
- ☐ rural
- ☐ urban
- ☐ greater city

30. I work as a school nurse in the following municipality :

©Copy rights reserved Maria Grandahl, Uppsala University. Please cite Grandahl et al. when using the questionnaire.

UPPSALA
UNIVERSITET

31. Years as a school nurse:

32. Years of experience working with HPV vaccination:_________

33. I am a Specialist Nurse in:

- ☐ pediatric nursing
- ☐ school health nursing
- ☐ district (primary care nurse)
- ☐ other, please specify
- ☐ I am not a Specialist Nurse (I am a Reg Nurse, Bachelor)

34. I mainly vaccinate children against HPV in

- ☐ Middle school
- ☐ Lower secondary school
- ☐ Upper secondary school

35. Number of children I have vaccinated the past year (approximately)

- ☐ None
- ☐ ≤10
- ☐ 11-20
- ☐ 21-40
- ☐ 41-60
- ☐ 60-80
- ☐ >80

Thank you for your reply!

©Copy rights reserved Maria Grandahl, Uppsala University. Please cite Grandahl et al. when using the questionnaire.
